# Effectiveness of ^18^F-FDG PET/CT in finding lung metastasis from a retroperitoneal paraganglioma

**DOI:** 10.22038/AOJNMB.2023.74066.1516

**Published:** 2024

**Authors:** Tomonori Chikasue, Seiji Kurata, Shuji Nagata, Shuichi Tanoue, Akiko Sumi, Mizuki Gobaru, Toru Hisaka, Toshihiro Hashiguchi, Takuya Furuta, Jun Akiba, Kiminori Fujimoto, Toshi Abe

**Affiliations:** 1 Department of Radiology, Kurume University School of Medicine, Kurume, Japan; 2 Division of Endocrinology and Metabolism, Department of Internal Medicine, Kurume University School of Medicine, Kurume, Japan; 3 Department of Surgery, Kurume University School of Medicine, Kurume, Japan; 4 Department of Diagnostic Pathology, Kurume University Hospital, Kurume, Japan

**Keywords:** Paraganglioma, Retroperitoneal tumor, Lung metastasis, ^ 123^I-MIBG scintigraphy, ^ 18^F-FDG PET/CT

## Abstract

A 50-year-old woman was diagnosed with iron deficiency anemia on general medical examination. Further, contrast-enhanced abdominal CT and magnetic resonance imaging revealed a large hypervascular mass with internal degeneration and necrosis in the retroperitoneal space. She was referred to our hospital for further evaluation and treatment. Because the paraganglioma was most likely as the imaging diagnosis, ^123^I-MIBG scintigraphy was performed. It revealed the marked abnormal accumulation in the retroperitoneal lesion indicating the paraganglioma and no other abnormal accumulation was noted. Several plasma catecholamines and their urinary metabolites were normal. On the subsequent ^18^F-FDG PET/CT, high FDG uptake was found in the retroperitoneal lesion (SUV_max_=38). FDG uptake was also found in a small nodule at the base of the lower lobe of the right lung (SUV_max_= 9.8). Contrast-enhanced imaging revealed a hypervascular nodule at the base of the right lung, suggesting pulmonary metastasis of a paraganglioma. The abdominal lesion and right lung nodule were excised, and retroperitoneal paraganglioma and pulmonary metastasis were diagnosed based on the pathology findings. In this case, ^18^F-FDG PET/CT was useful in the search for paraganglioma metastasis. We report a relationship between ^123^I-MIBG accumulation and ^18^F-FDG uptake in paraganglioma and review the relevant literature.

## Introduction

 Paraganglioma (PG) is a rare neuroendocrine tumor that arises from the extra-adrenal neuro-ectoderm. Metastases develop in approximately 14%–50% of PG cases (1). ^18^F- fluorodeoxyglucose (FDG) has been reported as more sensitive than ^123^I-metaiodobenzyl-guanidine (MIBG) in detecting malignant pheochromocytoma/PG (PPGL) (2). 

 Here, we report our experience with a case of pulmonary metastasis from PG not detected by ^123^I-MIBG scintigraphy but diagnosed through ^18^F-FDG positron emission tomography/ computed tomography (PET/CT).

## Case report

 A woman in her 50s was aware of an abdominal mass but had neglected it for a month. During a medical check-up with her general practitioner, she was diagnosed with the iron deficiency anemia. Subsequent contrast-enhanced abdominal CT and magnetic resonance imaging (MRI) revealed a retroperitoneal mass with a maximum diameter of approximately 10 cm. The patient was referred to our gastroenterology center for the further investigation and management.

 At the time of presentation to our hospital, the woman had no abnormal vital signs. Blood tests revealed only mild iron deficiency anemia and no other abnormal findings.

**Figure 1:**
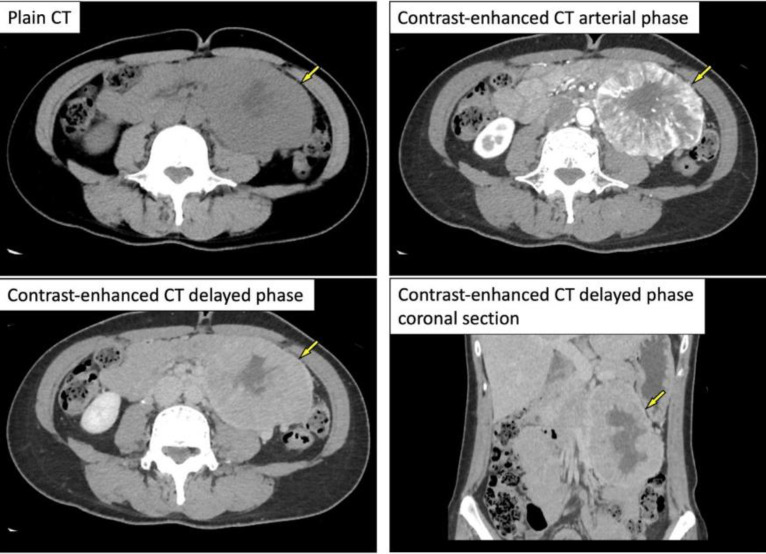
Contrast-enhanced computed tomography (**CT**) revealing an 8 × 7 × 12cm^3^ mass lesion in the left retroperitoneal space (⇨). Dynamic CT revealing high-contrast enhancement in the arterial phase and washout in the delayed phase

**Figure 2:**
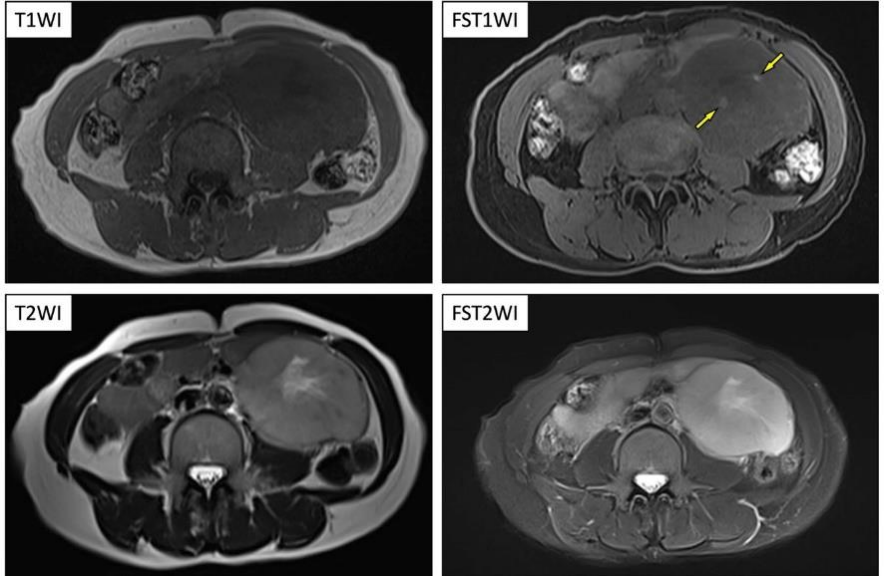
Magnetic resonance imaging showing low-signal lesions on T1-weighted imaging (**T1WI**) and high-signal lesions on T2-weighted imaging (**T2WI**). A signal change in the center of the lesion suggested degeneration or necrosis can be observed. Fat-suppressed T1WI (**FST1WI**) revealing a faint high-signal area within the lesion (⇨)—a finding suspicious for internal haemorrhage. **FST2WI**, fat-suppressed T2WI

 Contrast-enhanced CT presented an 8×7×12 cm^3^ mass lesion in the left retroperitoneal space. Dynamic CT study showed high-contrast enhancement in the arterial phase and washout in the late phase (Figure 1). The intratumoral signal presented low-signal on T1-weighted images and high-signal intensity on T2-weighted images. Signal changes indicating the degeneration or necrosis were also observed in the center of the lesion, as shown on CT. Fat-suppressed T1-weighted images showed a faint high-signal area within the lesion, which was indicating the internal hemorrhage (Figure 2).

 These findings on CT and MRI supported the diagnosis as retroperitoneal PG with degeneration/necrosis and haemorrhage. 

**Figure 3:**
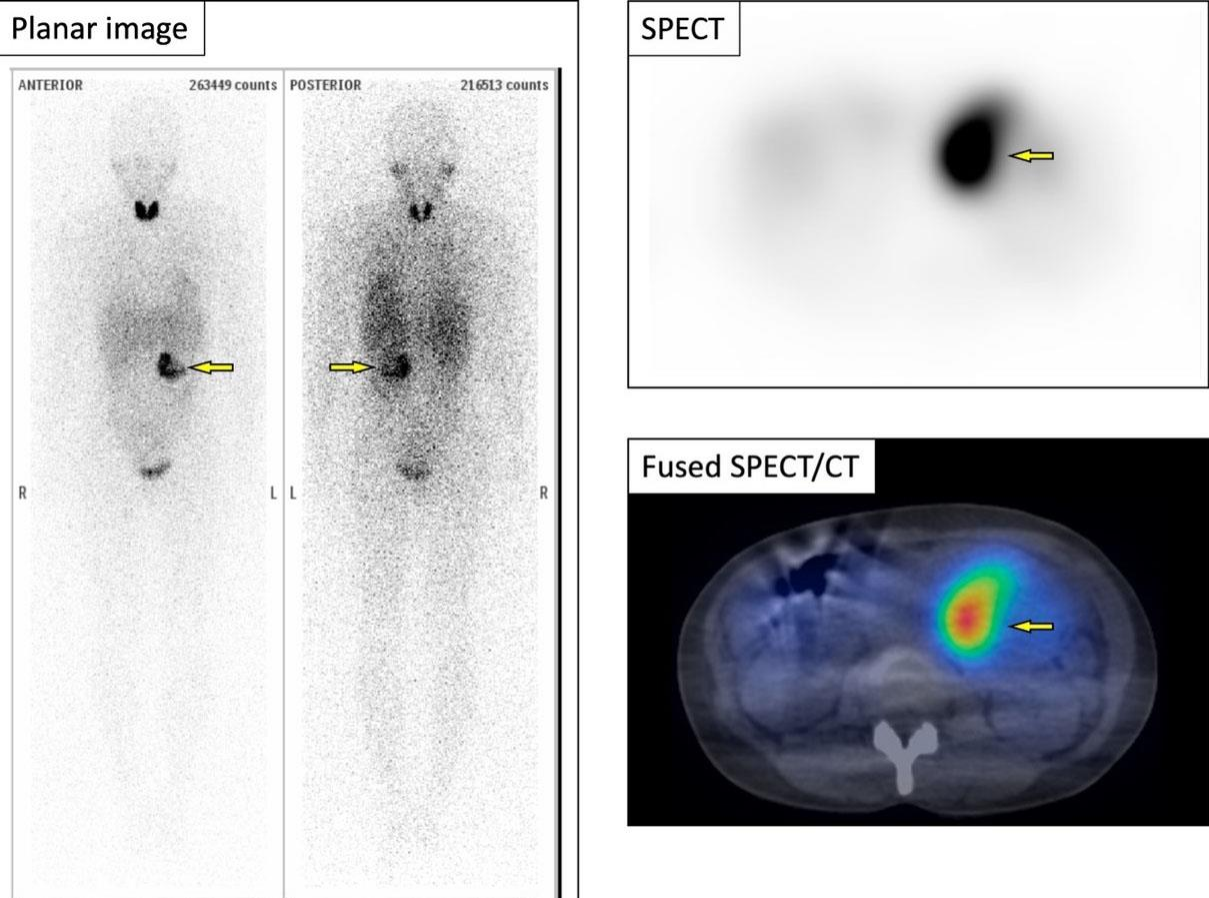
^123^I-MIBG scintigraphy revealing only one abnormal accumulation consistent with the retroperitoneal lesion (⇨). ^12^^3^I-MIBG accumulation is shown only in part of the retroperitoneal lesion. SPECT/CT, single photon emission CT/CT

 Subsequent ^123^I-MIBG scintigraphy revealed the solitary abnormal accumulation in the retroperitoneal lesion corresponding to the tumor; the finding was consistent with PG (Figure 3). ^123^I-MIBG accumulation is shown only in part of the retroperitoneal lesion. 

 Several plasma catecholamines and their urinary metabolites were normal.

**Figure 4:**
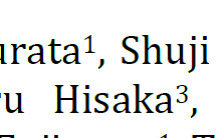
^18^F-FDG positron emission tomography/computed tomography (PET/CT) showing intense FDG uptake (maximum standardized uptake value [SUV_max_], 38) in the retroperitoneal lesion (⇨, ^18^F-FDG MIP image and fused PET/CT). FDG uptake in a small nodule at the base of the lower lobe of the right lung (SUV_max_, 9.8) is also observed (▻, ^18^F-FDG MIP image and fused PET/lung window CT). MIP, maximum intensity projection

**Figure 5:**
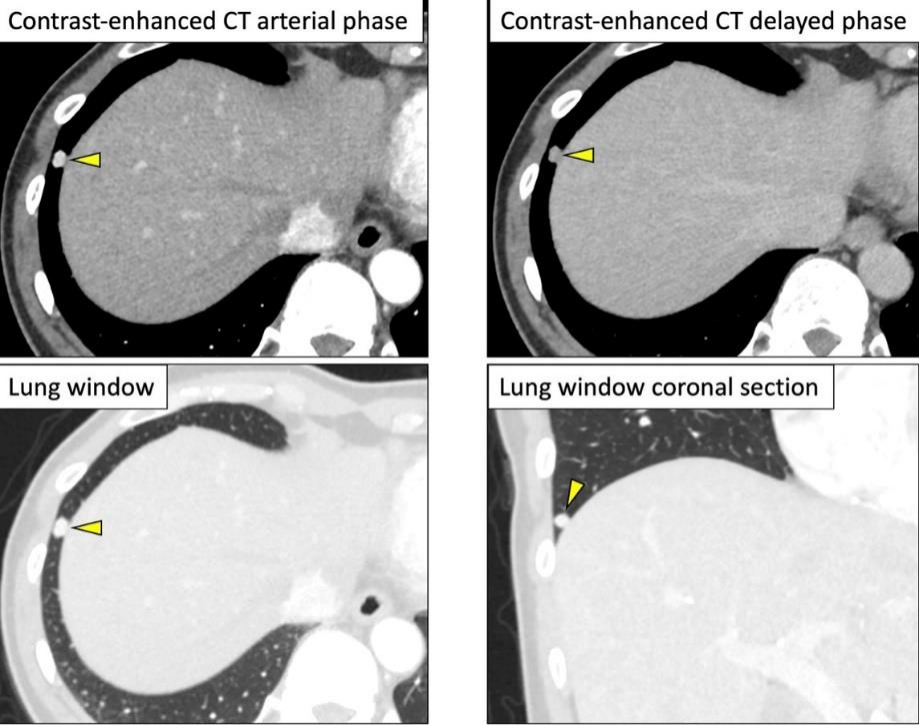
Contrast-enhanced CT showing a 0.7 × 0.5 × 0.7cm^3^ nodule in the right lung base that is strongly contrasted in the arterial phase and washed out in the delayed phase (▻)

^18^F-FDG PET/CT revealed high FDG uptake in the retroperitoneal lesion (maximum standardized uptake value [SUV_max_]=38). In addition, a small nodule-like FDG uptake was found at the base of the lower lobe of the right lung (SUV_max_=9.8) (Figure 4). By reviewing the contrast-enhanced dynamic CT, a 0.7×0.5×0.7 cm^3^ nodule was found at the base of the lower lobe of the right lung presenting high contrast-enahncement in the arterial phase and washed out in the late phase (Figure 5); this led us to suspect pulmonary metastasis of PG.

 The retroperitoneal lesion was subsequently resected. On gross examination, the split surface of the lesion was brownish in color. 

**Figure 6:**
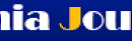
Split surface of the resected lesion showing brownish color upon gross examination. Histologically, the lesion comprised large polygonal cells with abundant cytoplasm. Abundant intervening blood vessels surrounded the cell cords and cytoplasm. The nuclei of the component cells are enlarged and irregular in shape. Immunostaining showing positive for chromogranin A and partially positive for S100; the MIB-1 index is 26.6%. HE, hematoxylin and eosin

 Histologically, the lesion comprised large polygonal cells with abundant cytoplasm. Abundant intervening blood vessels surrounded the cell cords and cytoplasm. The nuclei of the component cells were enlarged and irregular in shape. Immunostaining was positive for chromogranin A and partially positive for S100; the MIB-1 labeling index was 26.6% (Figure 6).

 Based on these findings, the tumor was determinately diagnosed as PG. Moreover, as the lesion had a score of 12 on the Pheochromocytoma of the Adrenal Gland Scoring Scale, it was considered a lesion with a malignant clinical course.

 The right pulmonary nodule was resected and its pathological findings was similar to that of the retroperitoneal lesion, leading to a diagnosis of pulmonary metastasis from the retroperitoneal PG. There has been no postoperative recurrence, and the patient is under observation at our hospital.

## Discussion

 PPGL is a rare tumor of the adrenal medulla and extra-adrenal sympathetic chromaffin tissues. PG is particularly rare, accounting for 0.3% of all tumors. Seventy-five percent of all PGs arise in the retroperitoneal and para-aortic regions (3, 4). Since the 5th edition of the WHO classification in 2022, PGs were subdivided into adrenal (pheochromocytoma) and extra-adrenal PG, based on the site of origin (5).

 PPGLs can be categorized as functional and nonfunctional. Functional PPGLs secrete epinephrine and norepinephrine. Patients with functional PPGLs clinically present with hypertension, headache, excessive sweating, hypermetabolism, and hyperglycemia. 

 Nonfunctional PPGLs are characterized by nonspecific symptoms related to tumor enlargement, including abdominal mass, epicardial discomfort, and nausea. These nonspecific symptoms are often the main complaints. Diagnosing a primary PPGL as benign or malignant is sometimes difficult based on pathology findings alone (6), therefore, malignant PPGL can be diagnosed based on the presence of metastasis to nonchromophilic tissues, such as liver, lungs, bones, and lymph nodes (7, 8). Metastasis occurs in 2.5%–14% of pheochromocytomas and 14%–50% of PGs (1).

 The features suggesting the malignant PPGLs include a tumor size of >5 cm, a score >6 on the Pheochromocytoma of the Adrenal Gland Scoring Scale, a Ki-67 labeling index of >3%, signal transducer and activator of transcription 3 (*STAT3*), heat-shock protein 90 (*HSP90*) and germline succinate dehydrogenase complex, subunit B (*SDHB*) gene mutation (9-11).

 In a previous report (2), the sensitivity of ^18^F-FDG in nonmetastatic PPGL was reportedly similar to that of ^123^I-MIBG (^18^F-FDG, 76.8%; ^123^I-MIBG, 75.0%); however, in metastatic disease, ^18^F-FDG was more sensitive (^18^F-FDG, 82.5%; ^123^I-MIBG, 50.0%). Tumor differentiation, tumor size, and genetic abnormalities are considered to be the cause of the negative ^123^I-MIBG accumulation in malignant PPGLs. Although it has been reported that 60% to 70% of PPGLs express noradrenergic transporters at the cell membrane, independent of hormonal activity (12), decreased expression of specific transporters due to dedifferentiation or other factors is reportedly involved in decreased ^123^I-MIBG accumulation, especially in metastatic or recurrent PGs (13-15). In this case also, ^123^I-MIBG was accumulated only in a part of the lesion, indicating that differences in transporter expression may have occurred within the lesion.

 Although ^123^I-MIBG accumulation is generally low in dedifferentiated tumor cells, high ^18^F-FDG uptake in tumor cells suggests the high proliferative potential due to the activated cellular energy metabolism. In cases where the diagnostic performance of ^123^I-MIBG is low, such as in malignant PPGL and *SDH* mutation, ^18^F-FDG PET has a high diagnostic value and can be used to evaluate metastases (2, 13, 14, 16). 

 With respect to the relationship between malignant PPGL and ^18^F-FDG uptake, a previous report suggested that testing for *SDH* and *VHL* mutation should be prioritized for cases with SUV exceeding 5 (2).

 In the present case, we did not examine genetic abnormalities in the primary lesion nor the lung metastasis, but we speculate that lung metastasis was negative for ^123^I-MIBG accumulation because of its small size. Another possibility is that the spatial resolution of the Single Photon Emission Computed Tomography/ CT used for ^123^I-MIBG scintigraphy was 10 mm, which could have failed to detect the lung metastasis for lesions <10 mm in size. From the experience of the present case, we suggest that ^18^F-FDG PET/CT should be performed to survey the metastasis when PPGL is detected.

 Informed consent is obtained from the patient regarding the case report and complies with the 1964 Declaration of Helsinki (https:// www.wma.net/policies-post/wma-declaration- of-helsinki-ethical-principles-for-medical-research-involving-human-subjects/) and subsequent revisions.

## References

[1] Choi YM, Sung TY, Kim WG, Lee JJ, Ryu JS, Kim TY (2015). Clinical course and prognostic factors in patients with malignant pheochromocytoma and paraganglioma: A single institution experience. J Surg Oncol..

[2] Timmers HJ, Chen CC, Carrasquillo JA, Whatley M, Ling A, Eisenhofer G (2012). Staging and functional characterization of pheochromo-cytoma and paraganglioma by 18F-fluorodeoxyglucose (18F-FDG) positron emission tomography. J Natl Cancer Inst..

[3] Lau D, La Marca F, Camelo-Piragua S, Park P (2013). Metastatic paraganglioma of the spine: case report and review of the literature. Clin Neurol Neurosurg..

[4] Fries JG, Chamberlin JA (1968). Extra-adrenal pheochromocytoma: Literature review and report of a cervical pheochromocytoma. Surgery..

[5] WHO Classification of Tumours Editorial Board Endocrine and Neuroendocrine tumours, vol. 8.

[6] Mikhail RA, Moore JB, Reed DN Jr, Abbott RR (1986). Malignant retroperitoneal para-gangliomas. J Surg Oncol..

[7] Lam AK (2017). Update on Adrenal Tumours in 2017 World Health Organization (WHO) of Endocrine Tumours. Endocr Pathol..

[8] Huang KH, Chung SD, Chen SC, Chueh SC, Pu YS, Lai MK (2007). Clinical and pathological data of 10 malignant pheochromocytomas: long-term follow up in a single institute. Int J Urol..

[9] Angelousi A, Kassi E, Zografos G, Kaltsas G (2015). Metastatic pheochromocytoma and para-ganglioma. Eur J Clin Invest..

[10] Thompson LD (2002). Pheochromocytoma of the Adrenal gland Scaled Score (PASS) to separate benign from malignant neoplasms: a clinicopathologic and immunophenotypic study of 100 cases. Am J Surg Pathol..

[11] Kulkarni MM, Khandeparkar SG, Deshmukh SD, Karekar RR, Gaopande VL, Joshi AR (2016). Risk Stratification in paragangliomas with PASS (Pheochromocytoma of the Adrenal Gland Scaled Score) and immunohistochemical markers. J Clin Diagn Res..

[12] Tan TH, Hussein Z, Saad FF, Shuaib IL (2015). Diagnostic Performance of (68)Ga-DOTATATE PET/CT, (18)F-FDG PET/CT and (131)I-MIBG Scintigraphy in Mapping Metastatic Pheochromocytoma and Paraganglioma. Nucl Med Mol Imaging..

[13] Fottner C, Helisch A, Anlauf M, Rossmann H, Musholt TJ, Kreft A (2010). 6–18F-fluoro-L-dihydroxyphenylalanine positron emission tomography is superior to 123I-meta-iodobenzyl-guanidine scintigraphy in the detection of extraadrenal and hereditary pheochromocytomas and paragangliomas: correlation with vesicular monoamine transporter expression. J Clin Endocrinol Metab..

[14] Fonte JS, Robles JF, Chen CC, Reynolds J, Whatley M, Ling A (2012). False-negative ¹²³I-MIBG SPECT is most commonly found in SDHB-related pheochromocytoma or paraganglioma with high frequency to develop metastatic disease. Endocr Relat Cancer..

[15] Van Berkel A, Pacak K, Lenders JW (2014). Should every patient diagnosed with a phaeochromocytoma have a ¹²³I-MIBG scintigraphy?. Clin Endocrinol (Oxf)..

[16] Gimenez-Roqueplo AP, Caumont-Prim A, Houzard C, Hignette C, Hernigou A, Halimi P (2013). Imaging work-up for screening of paraganglioma and pheochromocytoma in SDHx mutation carriers: a multicenter prospective study from the PG EVA Investigators. J Clin Endocrinol Metab..

